# Nickel–vanadium monolayer double hydroxide for efficient electrochemical water oxidation

**DOI:** 10.1038/ncomms11981

**Published:** 2016-06-16

**Authors:** Ke Fan, Hong Chen, Yongfei Ji, Hui Huang, Per Martin Claesson, Quentin Daniel, Bertrand Philippe, Håkan Rensmo, Fusheng Li, Yi Luo, Licheng Sun

**Affiliations:** 1 Department of Chemistry, Organic Chemistry, KTH Royal Institute of Technology, 10044 Stockholm, Sweden; 2 State Key Laboratory of Advanced Technology for Materials Synthesis and Processing, Wuhan University of Technology, Wuhan 430070, China; 3 Division of Theoretical Chemistry and Biology, School of Biotechnology, KTH Royal Institute of Technology, SE-106 91 Stockholm, Sweden; 4 Department of Chemistry, Surface and Corrosion Science, KTH Royal Institute of Technology, SE-10044 Stockholm, Sweden; 5 Department of Physics and Astronomy, Uppsala University, Box 516, 751 20 Uppsala, Sweden; 6 State Key Laboratory of Fine Chemicals, DUT-KTH Joint Education and Research Center on Molecular Devices, Dalian University of Technology (DUT), Dalian 116024, China

## Abstract

Highly active and low-cost electrocatalysts for water oxidation are required due to the demands on sustainable solar fuels; however, developing highly efficient catalysts to meet industrial requirements remains a challenge. Herein, we report a monolayer of nickel–vanadium-layered double hydroxide that shows a current density of 27 mA cm^−2^ (57 mA cm^−2^ after ohmic-drop correction) at an overpotential of 350 mV for water oxidation. Such performance is comparable to those of the best-performing nickel–iron-layered double hydroxides for water oxidation in alkaline media. Mechanistic studies indicate that the nickel–vanadium-layered double hydroxides can provide high intrinsic catalytic activity, mainly due to enhanced conductivity, facile electron transfer and abundant active sites. This work may expand the scope of cost-effective electrocatalysts for water splitting.

## Abstract (web summary)

Water oxidation is a key component of water splitting, but more effective catalysts for the process are still required. Here the authors synthesize a monolayer nickel-vanadium layered double hydroxide material showing high activity for water oxidation owing to enhanced conductivity and abundant active sites.

## Graphical abstract

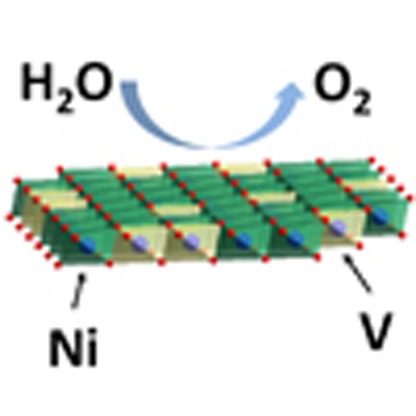

Water splitting is considered one of the most promising strategies to produce chemical fuels such as hydrogen. The half reaction of the water splitting process, water oxidation, remains the bottleneck of the whole process at present. Therefore, developing highly efficient water oxidation catalysts is crucial. Some precious metal-based electrocatalysts, such as IrO_2_ and RuO_2_, have shown excellent performance for water oxidation; however, they suffer from high-cost and relative scarcity of precious metals, which limits their applications. Although some first-row transition metal oxides (for example, NiO_x_, NiFeO_x_, CoO_x_ and MnO_x_) had been developed as low-cost electrocatalysts for water oxidation, most of them still cannot compete with IrO_2_ and RuO_2_^1^,^2^. Recently, the earth-abundant Ni–Fe double-layered hydroxide (NiFe-LDH) catalysts have attracted attention^3^,^4^,^5^,^6^,^7^. From being first reported as an advanced electrocatalyst coupled with carbon nanotubes for water oxidation^8^, it is nowadays known as one of the most active catalysts with a low overpotential and high electrolysis current. Since, then tremendous efforts have been devoted to further improve the activity of NiFe-LDH, such as exfoliation^9^ and hybridization^6^,^8^,^10^,^11^, to the extent that LDH catalysts can now outperform IrO_2_ in alkaline media^6^,^8^,^9^; however, the aforementioned methods are still too complicated for large-scale applications.

It is already known that Fe(III) incorporated in Ni(II)-based LDH is the key aspect for the high catalytic performance, although the role of Fe in LDH is still ambiguous^5^,^6^,^12^. Besides Fe(III), cobalt is also commonly incorporated in nickel hydroxides to construct NiCo-LDHs for water oxidation^13^,^14^,^15^,^16^, and the resulting NiCo-LDHs show promising catalytic activities; however, the performance is relatively low compared with the reported NiFe-LDHs under identical conditions^9^. Besides, more earth-abundant metal elements have been incorporated into Ni(OH)_2_ to explore novel LDHs for water oxidation, for example, recently Koper and co-workers^17^ investigated a series of Ni-based double hydroxides with Cr, Mn, Fe, Co, Cu and Zn for water oxidation, and among these candidates NiFe-LDH still appears as the most promising and shows the best activity. Up until now, there has been no reported earth-abundant metal element that can outperform Fe incorporated Ni-based LDHs. Searching for an earth-abundant metal to form efficient Ni-based LDH comparable to NiFe-LDH is still the state-of-the-art in this area of energy research.

In this work, we incorporate another earth-abundant element into Ni(OH)_2_: vanadium, and succeed in forming NiV-LDH as an efficient catalyst for the water oxidation reaction. A simple one-step hydrothermal method is employed to synthesize NiV-LDH. Without need for exfoliation or hybridization with other materials, the resulting monolayer NiV-LDH catalyst exhibits comparable activity to the best-performing NiFe-LDH for water oxidation in alkaline electrolyte.

## Results

### Monolayer of Ni_0.75_V_0.25_-LDH

**Figure 1:**
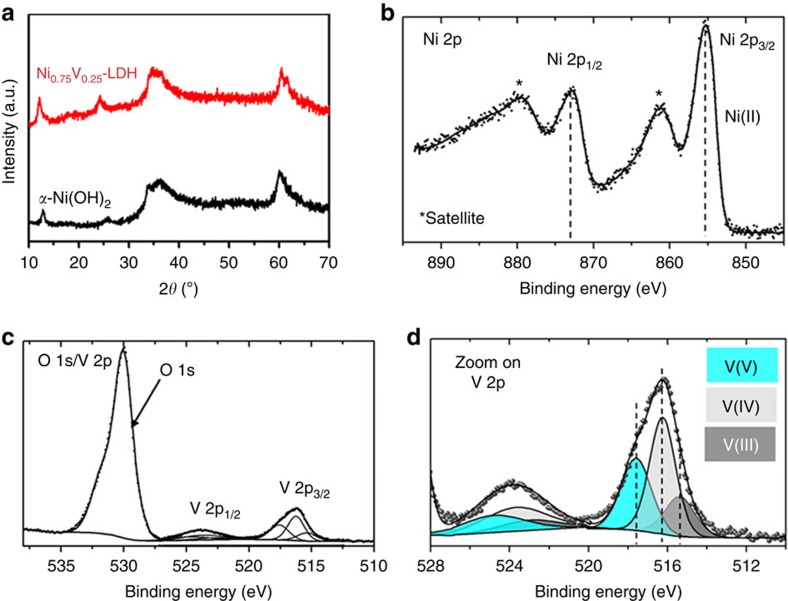
X-ray diffraction and XPS. (**a**) X-ray diffraction patterns of α-Ni(OH)_2_ and Ni_0.75_V_0.25_-LDH. XPS measurements (*hν*=1,486.6 eV) on the Ni_0.75_V_0.25_-LDH powder deposited on a FTO conductive glass: (**b**) Ni 2*p* (**c**) O 1*s* and V 2*p* core-level spectra, and (**d**) zoom on the V 2*p* core-level spectrum.

Figure 1a shows the typical X-ray diffraction patterns of pure Ni(OH)_2_ and Ni_0.75_V_0.25_-LDH. As can be seen, pure Ni(OH)_2_ was successfully synthesized in the simple hydrothermal system without addition of V sources, and the X-ray diffraction pattern supports the formation of pure hexagonal α-Ni(OH)_2_ (JCPDS 380715), which exhibits a layered structure constructed from [NiO_6_] coordinated octahedra connected by sharing their edges. After incorporation of V into the structure of α-Ni(OH)_2_, no obvious change can be observed in the X-ray diffraction spectrum, indicating Ni_0.75_V_0.25_-LDH is the isomorphous compound as α-Ni(OH)_2_ with the layered structure. The X-ray diffraction pattern of prepared bare V-based hydroxide without Ni source (Supplementary Fig. 1) shows very low crystallinity that is significantly different from those of the above Ni-based materials. Figure 1b–d shows the Ni 2*p*, V 2*p* and O 1*s* X-ray photoelectron spectroscopy (XPS) core-level spectra of the Ni_0.75_V_0.25_-LDH powder. The Ni 2*p* spectrum presents two main structures, resulting from the spin–orbit splitting of the *p* orbital that are assigned as Ni 2*p*_3/2_ (850−870 eV region) and Ni 2*p*_1/2_ (870−890 eV region). The binding energy difference between the 3/2 and 1/2 components is *∼*17.4 eV. Ni 2*p*_3/2_ presents a main peak at *∼*855.4 eV with an intense satellite structure at *∼*861.2 eV, (*∼*872.8 and *∼*879.6 eV, respectively, for Ni2*p*_1/2_), this signature is characteristic of Ni^2+^ (refs 18, 19, 20, 21). The spectra of O 1*s* and V 2*p* are presented in Fig. 1c, the O 1*s* signal originates from Ni_0.75_V_0.25_-LDH, but its main contributions is from the fluorine doped tin oxide (FTO) glass substrate used with an O1*s*-binding energy ∼530 eV. The V 2*p* core-level spectrum is also decomposed into V 2*p*_3/2_ and V 2*p*_1/2_ due to the spin–orbit splitting that are separated by *∼*7.5 eV. A closer view on the V 2*p*_3/2_ region is shown in Fig. 1d. Three components can be distinguished at *∼*515.4 eV (in blue), *∼*516.3 eV (in light grey) and *∼*517.5 eV (in grey) and are in good agreement with respectively V^3+^, V^4+^ and V^5+^ (refs 22, 23, 24). This result indicates that V^3+^ was partially oxidized by oxygen to V^4+^ and V^5+^ during the synthesis.

**Figure 2:**
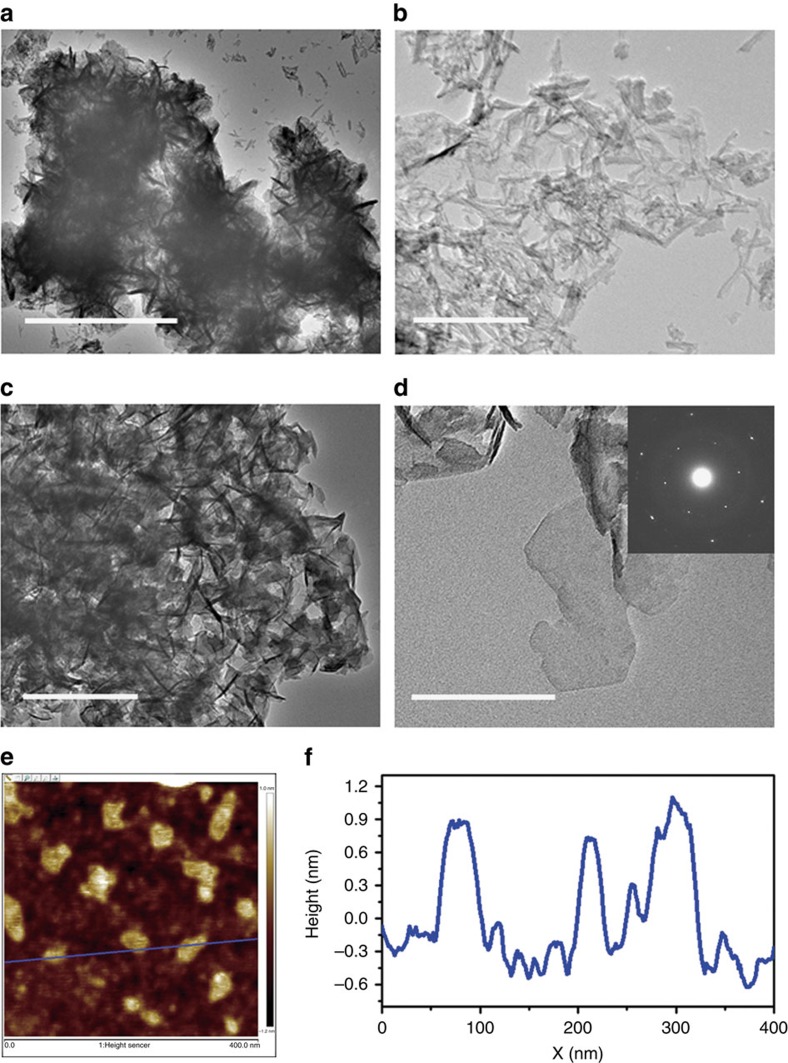
TEM and AFM. (**a**,**b**) TEM images of α-Ni(OH)_2_. Scale bar, 1 μm (**a**); 200 nm (**b**); (**c**,**d**) TEM images of Ni_0.75_V_0.25_-LDH (inset of **d**: selected area electron diffraction pattern). Scale bar, 200 nm (**c**); 100 nm (**d**); (**e**) AFM image and (**f**) height profile of Ni_0.75_V_0.25_-LDH nanosheets.

Typical transmission electron microscopy (TEM) images of *α*-Ni(OH)_2_ and Ni_0.75_V_0.25_-LDH are shown in Fig. 2. Interestingly, as shown in Fig. 2a,b, the pure *α*-Ni(OH)_2_ shows a narrow nanosheet morphology with the size of few tens of nanometre. Aggregated by these nanosheet crystals, a porous sphere structure with the diameter of few micrometres was obtained, as observed by scanning electron microscopy (SEM; Supplementary Fig. 2a). After adding the vanadium source to the initial solution for hydrothermal reaction, Ni_0.75_V_0.25_-LDH presents a three-dimensional morphology assembled by ultrathin nanosheets as shown in Fig. 2c,d. Selected area electron diffraction pattern in the inset of Fig. 2d confirms the hexagonal phase of Ni_0.75_V_0.25_-LDH. The atomic force microscopy (AFM) and height profile of Ni_0.75_V_0.25_-LDH in Fig. 2e,f show that the nanosheet is ultrathin with thickness of ∼0.9 nm, indicating the obtained nanosheet is monolayered. To make a comparison, Ni_0.75_Fe_0.25_-LDH was also prepared by a similar protocol in the literature^25^ and the SEM image of the products obtained is shown in Supplementary Fig. 2b and c. Some large aggregation plates in Ni_0.75_Fe_0.25_-LDH can be observed, showing that the size of aggregations by nanosheets in Ni_0.75_Fe_0.25_-LDH is bigger than Ni_0.75_V_0.25_-LDH. However, the TEM, AFM images and height profile of Ni_0.75_Fe_0.25_-LDH in Supplementary Fig. 2d–f exhibit that the nanosheet of Ni_0.75_Fe_0.25_-LDH is also ultrathin with thickness of ∼1.2 nm. Meanwhile, differing from the above nanosheet-based structure, the bare V-based and Fe-based hydroxides (β-FeOOH) without Ni content show mainly ‘nanostick' morphologies after the hydrothermal processes (Supplementary Fig. 3).

### Oxygen evolution catalysis

**Figure 3:**
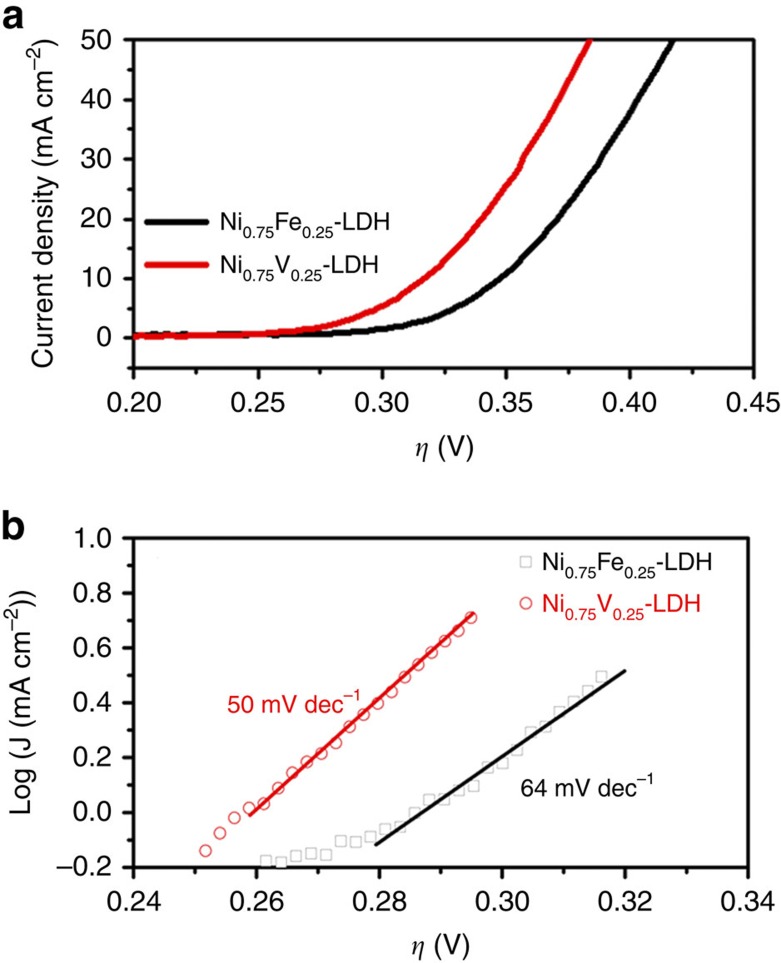
Linear scan voltammogram (LSV) curves and Tafel plots. (**a**) LSV curves and (**b**) Tafel plots (with ohmic-drop correction) of Ni_0.75_Fe_0.25_-LDH and Ni_0.75_V_0.25_-LDH.

It is known that the composition can affect the water oxidation performance of the catalysts significantly. First, we investigated the electrocatalytic activities of NiV-LDHs and NiFe-LDHs on glassy carbon (GC) electrodes with different Ni content to optimize the composition (the measurements were carried out without ohmic-drop correction unless noted otherwise). All the amount of catalyst loadings on GC electrodes were 0.143 mg cm^−2^. As shown in Supplementary Fig. 4, the Ni content plays an essential role for the catalytic activity in this study (the electrocatalytic activities of pure Ni(OH)_2_, bare V-based and Fe-based hydroxides are very low, so they are not shown here for clarity). When the molar ratio of Ni/X (X=V or Fe) is 3:1, that is, Ni_0.75_V_0.25_-LDH and Ni_0.75_Fe_0.25_-LDH, the best water oxidation performances are achieved with the highest catalytic current density (Supplementary Fig. 4a–c) and turnover frequency (TOF) (Supplementary Fig. 4d), and the lowest required overpotential (Supplementary Fig. 4e) in NiV-LDH and NiFe-LDH series, respectively (note that for NiFe-LDH, this optimized molar ratio of Ni/Fe is in a good agreement with the literature reported previously^6^). Either a lower or higher mole ratio of the two metal elements will decrease the catalytic performances of LDHs. The atomic percentage of Ni, V and Fe in Ni_0.75_V_0.25_-LDH and Ni_0.75_Fe_0.25_-LDH has been measured by energy-dispersive X-ray spectroscopy (EDS; Supplementary Fig. 5), showing the molar ratios of Ni/V (3.29) and Ni/Fe (2.93) are close to 3:1 that was used in the corresponding solution stoichiometries. The catalytic performances of Ni_0.75_V_0.25_-LDH and Ni_0.75_Fe_0.25_-LDH are very reproducible (Supplementary Fig. 6) and compared in Fig. 3. A sharp onset catalytic current density can be observed at low overpotential *η*∼250 and 300 mV for Ni_0.75_V_0.25_-LDH and Ni_0.75_Fe_0.25_-LDH, respectively. It is apparent that Ni_0.75_V_0.25_-LDH exhibits a better catalytic activity than Ni_0.75_Fe_0.25_-LDH, suggesting that substituting Fe entirely by V in Ni-based LDH can improve the water oxidation performance in our case. The improved catalytic activity of Ni_0.75_V_0.25_-LDH is not only shown via the higher catalytic current density of linear scan voltammogram (LSV) curves, but also reflected in its lower Tafel slope (Fig. 3b). Ni_0.75_V_0.25_-LDH catalyst exhibits a Tafel slope of ∼50 mV dec^−1^, which is smaller than that of Ni_0.75_Fe_0.25_-LDH catalyst (∼ 64 mV dec^−1^). Tafel slopes can be influenced by mass transport and electron transport^26^,^27^. Different scan rate ranging from 1 to 5 mV s^−1^ for LSV have been done, which resulted in negligible change of current densities for Ni_0.75_Fe_0.25_-LDH and Ni_0.75_V_0.25_-LDH catalysts, indicating sufficiently fast mass transport for both LDHs^26^,^27^. Therefore, it is reasonable to conclude that the lower Tafel slope of Ni_0.75_V_0.25_-LDH is likely ascribed to the facile electron transport through the layers of the catalyst.

At *η* of 350 mV, Ni_0.75_V_0.25_-LDH can achieve current density of 27.0±1.6 mA cm^−2^, which is more than twice higher than the one of Ni_0.75_Fe_0.25_-LDH (11.7±1.5 mA cm^−2^). TOF was also calculated at an overpotential of 350 mV in 1 M KOH, assuming all the metal sites were electrochemically active in the water oxidation reaction. Ni_0.75_V_0.25_-LDH also shows the better TOF of 0.054±0.003 s^−1^ compare with 0.021±0.003 s^−1^ for Ni_0.75_Fe_0.25_-LDH. It is worth noting that the above TOFs in this study are obviously underestimated, since some of the metal sites are electrochemically non-accessible. Meanwhile, the overpotential *η* required to achieve 10 mA cm^−2^ current density, which is approximately the current expected at the anode in a 10% efficient solar water splitting device under 1-sun illumination^28^, was also evaluated. Ni_0.75_Fe_0.25_-LDH and Ni_0.75_V_0.25_-LDH show required overpotential 0.347±0.002 and 0.318±0.003 V, respectively, exhibiting the lower required overpotential of Ni_0.75_V_0.25_-LDH.

**Figure 4:**
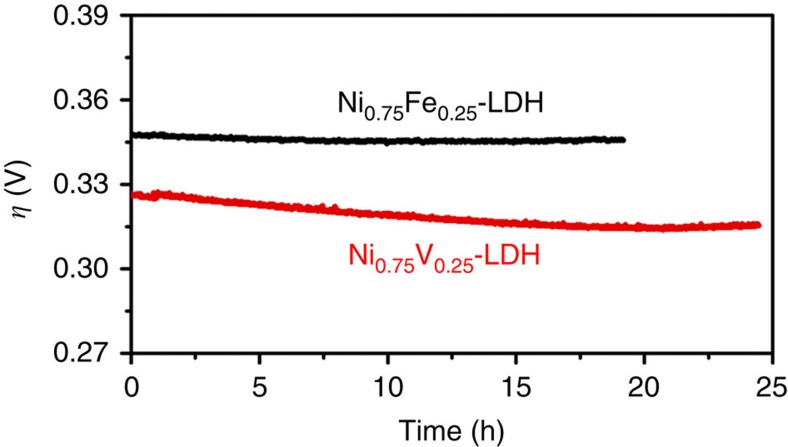
Long-term stability. Chronopotentiometry curves at current density of 10 mA cm^−2^ of Ni_0.75_Fe_0.25_-LDH and Ni_0.75_V_0.25_-LDH.

In addition, we compared the durability of Ni_0.75_Fe_0.25_-LDH and Ni_0.75_V_0.25_-LDH in 1 M KOH (Fig. 4). When biased galvanostatically at 10 mA cm^−2^, both of Ni_0.75_Fe_0.25_-LDH and Ni_0.75_V_0.25_-LDH electrodes show considerable stability, the slightly decrease of the required potential for Ni_0.75_V_0.25_-LDH electrode may be due to the amorphazation during the anodic conditioning process^26^,^29^. Obviously, Ni_0.75_V_0.25_-LDH electrode requires less overpotential than Ni_0.75_Fe_0.25_-LDH, showing its comparable stability and catalytic performance to Ni_0.75_Fe_0.25_-LDH for water splitting.

## Discussion

**Figure 5:**
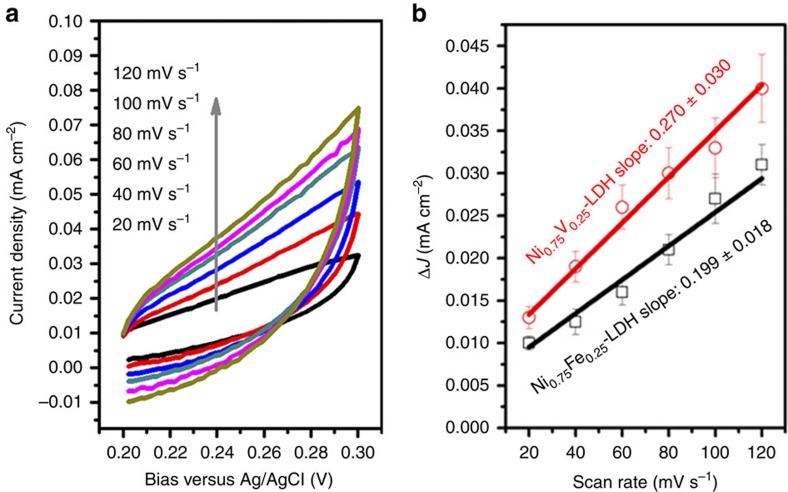
ECSA of Ni_0.75_Fe_0.25_-LDH and Ni_0.75_V_0.25_-LDH. (**a**) Typical cyclic voltammetry curves of Ni_0.75_V_0.25_-LDH electrode in 1 M KOH with different scan rates; (**b**) Δ*J* (=*J*_a_−*J*_c_) of Ni_0.75_Fe_0.25_-LDH and Ni_0.75_V_0.25_-LDH plotted against scan rates. All the error bars represent the s.d.'s of three replicate measurements. The slopes (2*C*_dl_) were used to represent ECSA. The unit of slopes is mF cm^−2^.

To understand the reason behind the high catalytic performance of Ni_0.75_V_0.25_-LDH for water splitting, electrochemical active surface areas (ECSA) of Ni_0.75_V_0.25_-LDH and Ni_0.75_Fe_0.25_-LDH were obtained from cyclic voltammetry (CV) curves in 1 M KOH and compared. Figure 5a shows typical CV curves of Ni_0.75_V_0.25_-LDH with different scan rates. By plotting the Δ*J*=(*J*_a_–*J*_c_) at 0.25 V versus Ag/AgCl against the scan rate, the linear slope that is twice the double layer capacitance (*C*_dl_) can be obtained, and is normally used to represent the corresponding ECSA^1^,^9^,^11^,^26^.

Active surface area is a very important factor for catalysts in water oxidation reaction, as it is well known that an increase of active surface area often leads to enhancement of the catalytic activity. The linear slope (ECSA) of Ni_0.75_Fe_0.25_-LDH is 0.199±0.018 mF cm^−2^, while Ni_0.75_V_0.25_-LDH has a higher linear slope of 0.270±0.030 mF cm^−2^, which means Ni_0.75_V_0.25_-LDH has a more electroactive surface than Ni_0.75_Fe_0.25_-LDH (Fig. 5b). The bigger ECSA should be attributed to the smaller size of aggregations by nanosheets in Ni_0.75_V_0.25_-LDH (as shown in Supplementary Fig. 2). This can contribute to partial enhancement of water oxidation reaction presented here. Nevertheless, it is important to note that comparing to Ni_0.75_Fe_0.25_-LDH, Ni_0.75_V_0.25_-LDH has just slightly ∼36% higher ECSA due to the similar nanostructure, with such small increase of ECSA, more than twofold of current density at *η*=350 mV can be achieved. This improvement of the catalytic activity cannot be merely ascribed to the slightly increased surface area of the catalysts. This result suggests that Ni_0.75_V_0.25_-LDH may have higher intrinsic catalytic activity for water oxidation reaction.

**Figure 6:**
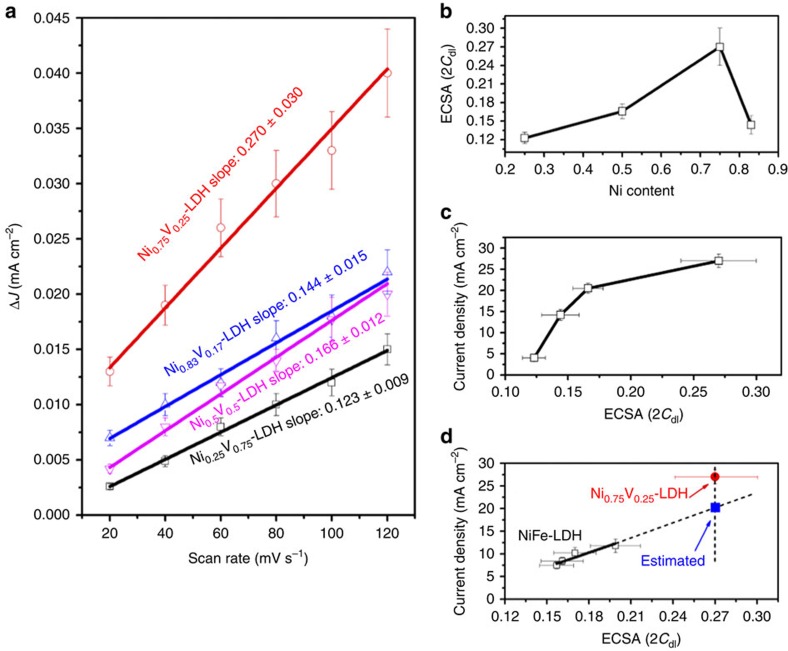
ECSA of LDHs. (**a**) Δ*J* (=*J*_a_−*J*_c_) of NiV-LDH with different Ni contents plotted against scan rates. The unit of slopes is mF cm^−2^; (**b**) ECSA (2*C*_dl_) of NiV-LDH with different Ni contents; (**c**) current density at 350 mV overpotential plotted against ECSA (2*C*_dl_) of NiV-LDH with different Ni contents; and (**d**) current density at 350 mV overpotential plotted against ECSA (2*C*_dl_) of NiFe-LDH with different Ni contents (black open squares), estimated the activity of NiFe-LDH (blue solid square) with the same ECSA of Ni_0.75_V_0.25_-LDH (red solid circle). All the error bars represent the s.d.'s of three replicate measurements.

To verify this point, the ECSAs of NiV-LDH with different Ni content were further examined in details, as shown in Fig. 6a–c. The slopes (ECSA) of Ni_0.25_V_0.75_-, Ni_0.5_V_0.5_-, Ni_0.75_V_0.25_- and Ni_0.83_V_0.17_-LDH electrodes were 0.123±0.009, 0.166±0.012, 0.270±0.030 and 0.144±0.015 mF cm^−2^, respectively, in Fig. 6a. Obviously, the largest ECSA can be achieved when the Ni content was 0.75 in NiV-LDHs, as shown in Fig. 6b as well. The relationship of ECSA with the current density of different NiV-LDHs is exhibited in Fig. 6c. One can see that the current density of NiV-LDH increases with ECSA, further confirming the active surface area of catalyst is an important contributor for the enhancement of water oxidation reaction. However, investigating this result carefully, the ECSA has more than twofold increase from ∼0.123 to 0.270 mF cm^−2^, while the corresponding current density increases from ∼4.0 to 27.0 mA cm^−2^, showing significantly almost seven times improvement. This is a strong clue that incorporating V into Ni hydroxide can not only change the active surface area, but also enhance the intrinsic catalytic property. As a comparison, we also investigated current density-dependent ECSA of different NiFe-LDHs in Fig. 6d. For NiFe-LDHs, the current density increases with ECSA as well; however, comparing with the significantly enhanced current density of NiV-LDH, the current density of NiFe-LDH just almost linearly increases to 1.6-fold (from ∼7.45 to 11.76 mA cm^−2^) with 1.3-fold increase of ECSA (from ∼0.157 to 0.199 mF cm^−2^). This linear correlation indeed indicates that the improvement of catalytic activity of NiFe-LDHs is mainly due to the increase of actively accessible surface area. We attempted to make a comparison of the catalytic activities between NiFe-LDH and NiV-LDH with the same ECSA value. The linear correlation in Fig. 6d for NiFe-LDH is used to predict the current density of NiFe-LDH with 0.270 mF cm^−2^ of ECSA (the blue solid square in Fig. 6d), which is supposed to have the same ECSA with Ni_0.75_V_0.25_-LDH catalyst (the red solid circle in Fig. 6d). The expected current density of NiFe-LDH described above (∼20.0 mA cm^−2^) is still lower than Ni_0.75_V_0.25_-LDH (∼27.0 mA cm^−2^), although they have been estimated to have the same ECSA value. These results suggest in addition to the increased accessible surface area, probably the V in the Ni-based LDHs can also lead to the increased intrinsic catalytic activity than NiFe-LDHs in our case.

Although it is difficult to correctly normalize the catalytic current to the number of active sites in the LDH electrocatalysts to compare the intrinsic catalytic performances accurately, the kinetic parameters of the Tafel slope and onset potential can be selected to reflect the intrinsic activity of catalysts^30^,^31^. Thus, we investigated the Tafel slopes and onset potentials of Ni_0.75_Fe_0.25_-LDH and Ni_0.75_V_0.25_-LDH in Fig. 3 again. The Tafel slope is only affected by the kinetics of the reaction, which involves the type of active sites but not its quantity, surface area, morphology or electrical resistance^30^. Therefore, the lower Tafel slope of Ni_0.75_V_0.25_-LDH (∼50 mV dec^−1^) than that of Ni_0.75_Fe_0.25_-LDH (∼64 mV dec^−1^) indicates the higher catalytic activity and superior type of the active sites in Ni_0.75_V_0.25_-LDH. In addition, Ni_0.75_V_0.25_-LDH also shows lower onset potential (∼250 mV overpotential) than that of Ni_0.75_Fe_0.25_-LDH (∼300 mV overpotential), implying the facile kinetics of Ni_0.75_V_0.25_-LDH for the water oxidation reaction, which is consistent with the Tafel slope investigation. These results indicate that Ni_0.75_V_0.25_-LDH possesses superior intrinsic catalytic activity in our case.

**Figure 7:**
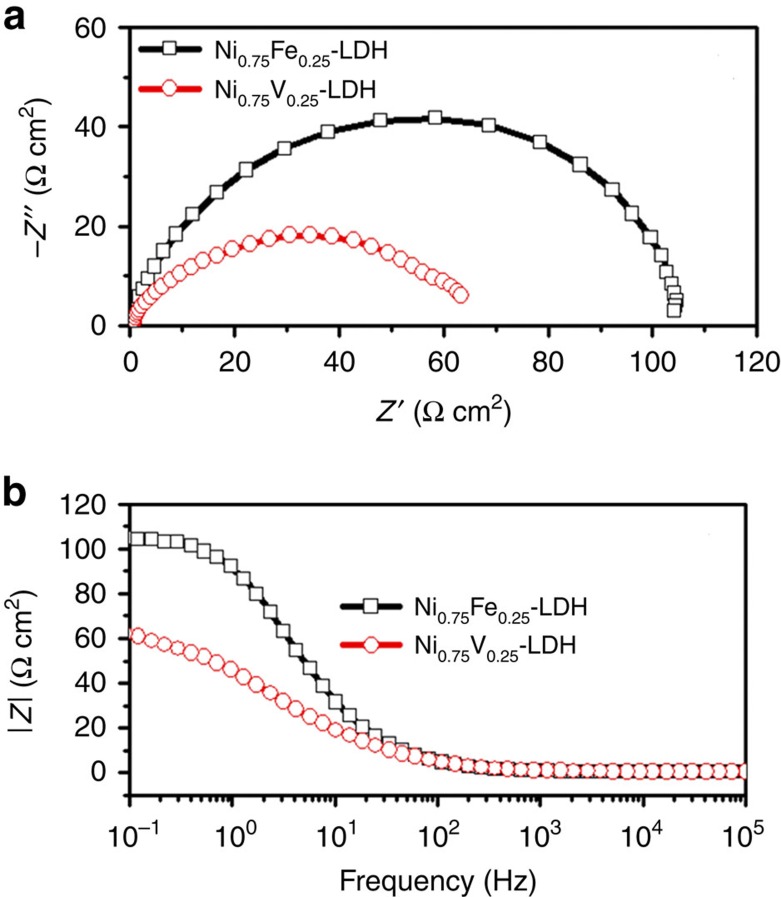
EIS measurements. (**a**) Nyquist diagram and (**b**) Bode plots of Ni_0.75_Fe_0.25_-LDH and Ni_0.75_V_0.25_-LDH with bias of 350 mV overpotential.

The higher intrinsic catalytic activity of Ni_0.75_V_0.25_-LDH may be due to the higher conductivity than Ni_0.75_Fe_0.25_-LDH. To test the conductivity, electrochemical impedance spectroscopies (EIS) of Ni_0.75_Fe_0.25_-LDH and Ni_0.75_V_0.25_-LDH electrodes were carried out in the three-electrode configuration in 1 M KOH. The Nyquist diagrams of both electrodes show an apparent semicircle in the high frequency range (Fig. 7a), which should be mainly associated with charge transfer resistance (*R*_ct_) in the LDH catalysts^32^,^33^. The diameter of the semicircle in Nyquist diagram of Ni_0.75_V_0.25_-LDH decreases comparing to that of Ni_0.75_Fe_0.25_-LDH, indicating lower charge transfer resistance, that is, improved conductivity in LDH catalysts. A reported electrical analogue was used to fit the EIS data^32^, and the *R*_ct_ of Ni_0.75_V_0.25_-LDH is estimated to be ∼62 Ω cm^2^, lower than that of Ni_0.75_Fe_0.25_-LDH (∼94 Ω cm^2^). This is also reflected in the Bode plots in Fig. 7b, where Ni_0.75_V_0.25_-LDH shows smaller resistance than Ni_0.75_Fe_0.25_-LDH. It is known that the LDHs suffer from their low conductivity, in the current case, the higher electron conductivity of Ni_0.75_V_0.25_-LDH benefits the charge transfer efficiently, which is also in a good agreement with the lower Tafel slope in Fig. 3b.

On the other hand, comparing to the relatively planar structure in Ni_0.75_Fe_0.25_-LDH, the slightly more three-dimensional structured Ni_0.75_V_0.25_-LDH can expose more edges of the nanosheets to the electrolyte (see TEM images in Fig. 2c,d and Supplementary Fig. 2). The edges are expected to contain open coordination sites that can be the active sites for water oxidation. Therefore, from this point, Ni_0.75_V_0.25_-LDH exposes more active sites than Ni_0.75_Fe_0.25_-LDH in electrolyte, while maintaining a similar active surface area. This may be another important factor contributing to the highly catalytic activity of Ni_0.75_V_0.25_-LDH. Meanwhile, investigating Fig. 6c again, it is also noted that the increases in ECSA produce large changes in current density up to ECSA=0.17 (2*C*_dl_), but after that the trend is much less pronounced. This change of trend of the activity dependent on ECSA for NiV-LDHs indicates that there should be other factors that can influence the activity of NiV-LDHs besides ECSA. It was reported that the catalytic performance of the layered catalyst for water splitting more strongly depends on the edge-state length than ECSA. The catalytic activity can be linear to the edge-state length^34^. Therefore, as shown in Fig. 6c, a plausible explanation is that it can be speculated that at small ECSA (<0.17 in our case), the active sites on the edge-state length can be exposed to the electrolyte sufficiently, increasing with ECSA, and dominate the catalytic activity, so the modest increases in ECSA can produce large changes in current density by the edge-state length. However, when the ECSA increases to a large value, due to the aggregation, ESCA will cover numerous edges of the nanosheets from the electrolyte, which makes ECSA show more significant effect than the edge-state length, resulting in a slower increase rate. This result also indicates the important role that the edge state of LDHs plays in the catalytic activity. More investigations into the detailed mechanism are in progress.

To further understand the advantages of Ni_0.75_V_0.25_-LDH, density functional theory (DFT) calculations were performed based on the following mechanism for water oxidation:


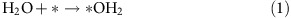



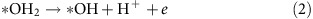



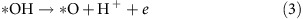



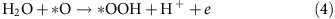



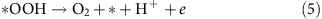


Here ‘*' presents the adsorption site that is usually on the top of the doping element. The calculation of the reaction free energy with the zero-point energy and entropy corrections followed the same procedure in ref. 35. Reaction free energies of reaction 1–5 are denoted as Δ*G*_1_–Δ*G*_5_, Δ*G*_5_ is defined as 4.92 eV–Δ*G*_1_–Δ*G*_2_–Δ*G*_3_–Δ*G*_4_ to avoid the calculation of the energy of O_2_ molecule. The overpotential *η* is defined in equation (1):


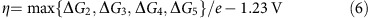


**Figure 8:**
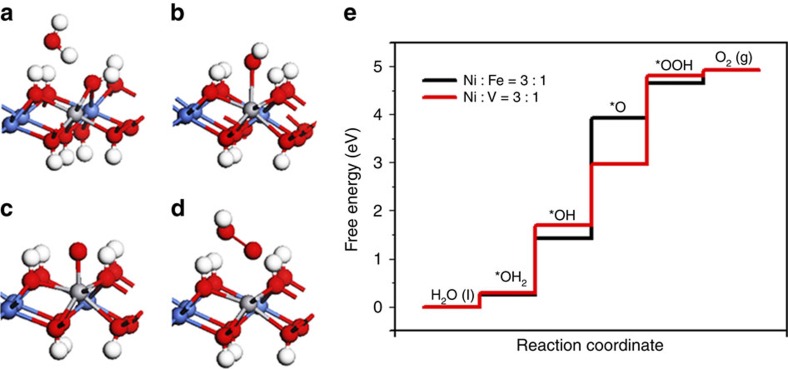
DFT calculation. Adsorption geometries of the intermediates H_2_O, *OH, *O and *OOH in **a**,**b**,**c** and **d**, respectively. The red, blue, white, grey atoms represent the O, Ni, H and V atoms, respectively. The adsorption structures are similar to these when one Ni is substituted by Fe instead of V; (**e**) the free-energy landscape.

The optimized structures of the intermediates in the free-energy landscape are shown in Fig. 8. The intermediate *OH and *OOH bind to the surface through oxygen with a single bond. The difference of their binding energies has been shown to be a constant (3.2±0.2 eV) for various materials, such as metals and oxides^36^. In our case, the constant is calculated to be 3.22 eV. Because this binding energy difference equals to the sum of the reaction free energies of reaction 3 and 4 (Δ*G*_3_+Δ*G*_4_)^35^,^37^, the rate-limiting step is either the formation of *O from *OH (Δ*G*_3_) or formation of *OOH from *O (Δ*G*_4_). The calculated free-energy landscape shows that the rate-limiting step is the formation of *OOH when the sample is doped with V with *η*=0.62 V, whereas the rate-limiting step becomes the formation of *O when the sample is doped with Fe with *η=*1.28 V. Because Δ*G*_3_+Δ*G*_4_=3.22 eV, the lower limit of the *η* can be reached when Δ*G*_3_=Δ*G*_4_=1/2 × 3.22 eV=1.61 eV if a proper element was doped. Then *η*=1.61–1.23=0.38 V, which suggests it has a large space to further optimize the activity of Ni-based LDH by doping with other elements.

Besides being better than the prepared NiFe-LDH in our case, Ni_0.75_V_0.25_-LDH shows superior catalytic performance than other reported LDHs as well, to the best of our knowledge. Supplementary Table 1 compares the activity of Ni_0.75_V_0.25_-LDH with recently reported state-of-the-art LDH catalysts without exfoliation or coupling with other materials, which were loaded on GC electrodes for water splitting, including NiFe-, NiCo-, ZnCo-, CoCo- and MnCo-LDHs. Although the catalytic performances of LDH electrodes are strongly dependent on the preparation methods in literatures, to make a general overview, the current density at 350 mV overpotential and the corresponding mass activity (A g^−1^) are still used to compare the intrinsic activities between different LDH catalysts. To make the comparison more reasonable, ohmic-drop correction was also performed for Ni_0.75_V_0.25_-LDH, as most of the literature has done (Supplementary Fig. 7)^8^,^9^,^26^,^29^,^38^. In our case, without ohmic-drop correction, at 350 mV overpotential, Ni_0.75_V_0.25_-LDH can achieve ∼27 mA cm^−2^ current density with ∼190 A g^−1^ mass activity. After ohmic-drop correction, the current density and mass activity can be as high as ∼57 mA cm^−2^ and ∼400 Ag^−1^, respectively. Those results are even better than some exfoliated NiFe-, NiCo- and CoCo-LDHs. It is worthy to note that Ni_0.75_V_0.25_-LDH shows even higher activity than MnCo-LDH (∼43 mA cm^−2^ and ∼301 A g^−1^) before long-term anodic conditioning that was reported as one of the most active LDH catalyst for water oxidation^26^.

It is worth pointing out that although Ni_0.75_V_0.25_-LDH outperforms Ni_0.75_Fe_0.25_-LDH in our case, we cannot claim Ni_0.75_V_0.25_-LDH has higher catalytic activity than all the NiFe-LDHs in the literature. For instance, Louie and Bell reported NiFe-LDH that requires <300 mV overpotential to deliver current densities of 10 mA cm^−2^ with a Tafel slope of ∼40 mV dec^−1^ (with ohmic-drop correction), which outperforms our Ni_0.75_V_0.25_-LDH probably due to the different preparation method^12^. Nevertheless, from Supplementary Table 1, our Ni_0.75_V_0.25_-LDH is still highly efficient among the listed catalysts, and comparable to the best-performing NiFe-LDHs^12^, which indicates our Ni_0.75_V_0.25_-LDH is one of the best water oxidation catalysts.

To confirm the O_2_ evolution and Faradaic efficiency of Ni_0.75_V_0.25_-LDH electrode, the experimental and theoretical O_2_ evolution amount by Ni_0.75_V_0.25_-LDH at a constant oxidative current of 1 mA in 1 M KOH were performed as shown in Supplementary Fig. 8. The experimental O_2_ evolution determined by gas chromatography exhibited 101±7% Faradaic efficiency, when the electrolysis time was 60 min. Moreover, to further explore the highly catalytic activity of Ni_0.75_V_0.25_-LDH, we drop-casted Ni_0.75_V_0.25_-LDH suspension onto Ni foam as well, which can supply excellent conductivity and very high surface area for the electrode, and tested the catalytic activity of resulted electrode in 1 M KOH for water oxidation. The Ni_0.75_V_0.25_-LDH on Ni foam exhibits the promising activity for practical applications, with ∼44 mA cm^−2^ at 350 mV overpotential, and just ∼300 mV overpotential required to reach 10 mA cm^−2^ (Supplementary Fig. 9) without ohmic-drop correction. Such catalytic activities of Ni_0.75_V_0.25_-LDH on Ni foam indicate attractive prospects for large-scale and practical applications.

In summary, Ni_0.75_V_0.25_-LDH has been prepared by a simple hydrothermal method, which is monolayered and shows high catalytic activity for the water oxidation reaction comparable to the best-performing NiFe-LDHs. The high intrinsic catalytic activity of Ni_0.75_V_0.25_-LDH is mainly due to the good conductivity, facile electron transfer and abundant active sites in the nanolayers of Ni_0.75_V_0.25_-LDH, showing potential to be one of the most effective Ni-based LDH electrocatalysts. Our study reveals the promising catalytic properties of vanadium incorporated Ni-based LDHs, and expands the scope of non-precious metal catalysts with the highly intrinsic activity for the water oxidation reaction.

## Methods

### Preparation of NiV- and NiFe-LDHs

A one-step hydrothermal method was employed to synthesize the bulk LDHs. In brief, for preparation of NiV-LDH, different mole ratios of Ni/V solution (1:0, 5:1, 3:1, 1:1, 1:3 and 0:1 for the synthesis of pure Ni(OH)_2_, Ni_0.83_V_0.17_-LDH, Ni_0.75_V_0.25_-LDH, Ni_0.5_V_0.5_-LDH, Ni_0.25_V_0.75_-LDH and bare V-based hydroxide, respectively) was obtained by mixing NiCl_2_ and VCl_3_ in 80 ml H_2_O, while the total amount of metal ions (Ni^2+^+V^3+^) was kept to 3.2 mmol. Afterwards, 0.3 g of urea was added, and the above mixture solution was transferred to a stainless-steel Teflon-lined autoclave, and heated in an oven at 120 °C for 12 h. After cooling the autoclave to room temperature, the resulting powder was washed by deionized water and ethanol three times, collected and then dried at 70 °C overnight. As a reference, for preparation of NiFe-LDH, the same processes were followed except FeCl_3_ was used as iron source instead of VCl_3_, which was analogous to the literature^25^.

### Electrode preparation

A measure of 5 mg of the obtained LDH powders were dispersed in the mixture solution of 1 ml H_2_O, 0.25 ml 2-propanol and 10 μl 5% Nafion (ethanol solution) by sonication for >1 h. A measure of 2.5 μl of the above suspension were drop-casted to a pre-polished GC electrode (diameter: 3 mm), and dried at 50 °C for 15 min to evaporate the solvent.

### Structure and surface characterization

X-ray diffraction measurements were carried out on Bruker X-ray diffraction diffractometer D5000 with Cu K*α* radiation. SEM images were taken on JEOL JSM-7000F instrument with energy-dispersive X-ray spectroscopy (EDS). TEM images were taken on JEOL JEM2100 TEM. Tapping mode AFM was performed to examine the surface morphology by a Dimension Icon AFM (Bruker, Santa Barbara, USA) on silicon wafer. Rectangular cantilevers with approximate dimensions of 125 μm in length and 40 μm in width (BudgetSensors Tap300Al-G) were used to perform the tapping mode experiments. The NanoScope Analysis software (version 1.50, Bruker) was used to analyse the recorded AFM data. A first-order polynomial-flattening algorithm was employed to remove surface tilt from height images. XPS measurements were conducted with an in-house spectrometer (PHI 5500) using monochromatized Al K*α* radiation (1,486.6 eV). The pressure in the analysis chamber was ∼5 × 10^−9^ mbar during the measurement. Core peaks were analysed using a nonlinear Shirley-type background. The peak positions and areas were optimized by a weighted least-squares fitting method using 70% Gaussian/30% Lorentzian lineshapes. The powder was grinded and deposited on a conductive FTO glass. An electron flood gun was used to compensate the charging effects. The XPS spectra were energy calibrated by setting the adventitious carbon peak to 285 eV.

### Electrochemical measurements

Electrolysis experiments were carried out in a polytetrafluoroethylene cell with an Autolab potentiostat with GPES electrochemical interface (Eco Chemie) in a standard three-electrode configuration, which was composed of working electrode (LDHs deposited on GC electrodes), counter electrode (Pt net) and reference electrode (Ag/AgCl). The electrolyte was 1 M KOH, and the applied potentials were converted with respect to reversible hydrogen electrode (RHE), *E*_RHE_=*E*_Ag/AgCl_+0.059 pH+0.197 V, and overpotential *η*=*E*_RHE_–1.23 V.

First, before all the electrochemical measurements, a galvanostatic measurement at a fixed current density of 5 mA cm^−2^ was performed until a stable potential was obtained. Then LSV were measured from 0.2 to 0.7 V versus Ag/AgCl with a slow scan rate of 2 mV s^−1^. By plotting overpotential *η* against log (*J*) from LSV curves, Tafel slopes can be obtained. To test the stability of LDHs, a galvanostatic measurement at a fixed current density (*J*) of 10 mA cm^−2^ was performed. ECSA were measured by CV at the potential window 0.2–0.3 V versus Ag/AgCl, with different scan rates of 20, 40, 60, 80, 100 and 120 mV s^−1^. By plotting the Δ*J*=(*J*_a_−*J*_c_) at 0.25 V versus Ag/AgCl against the scan rate, the linear slope that is twice of the double layer capacitance (*C*_dl_) is used to represent ECSA. All the above measurements were carried out without ohmic-drop correction unless noted otherwise.

### Turnover frequency

The TOF of LDH catalysts were calculated according to the following equation (2)^9^,


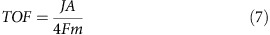


where *J* is the current density at a given overpotential, for example, in our cases *η*=350 mV, *A* is the surface area of the electrode (0.07 cm^2^), *F* is Faraday constant (96,485 s A mol^−1^) and *m* is the number of moles of the metal on the electrodes. In our cases, we assumed all the metal sites were actively involved in the electrochemical reaction.

To compare the conductivities of LDHs, EISs were carried out in 1 M KOH with a three-electrode configuration, the frequency ranged from 0.1 to 10^5^ Hz, with an a.c. amplitude of 10 mV and overpotential bias of 350 mV.

The measurements of O_2_ were performed in an air-tight H shape cell, which was divided by a glass frit to two chambers. The working electrode, the Ag/AgCl reference electrode and a magnetic stirring bar were inserted in one chamber of the cell, the Pt counter electrode was inserted in the other chamber. The cell was filled with 1 M KOH and degassed with helium for >1 h. The headspace of the compartment containing the working electrode was 23.6 ml. The electrolysis was carried out with a constant oxidation current of 1 mA for 60 min. A measure of 500 μl of the gas sample in the compartment containing the working electrode was transferred by a specific syringe to the gas chromatography, (Shimadzu) where the amount of O_2_ evolution was determined. The Faradaic efficiency was determined from the total amount of charge *Q* (*C*) passed through the cell and the total amount of the produced O_2_ *n*_O2_ (mol): Faradaic efficiency=4*F*** × ***n*_O2_/*Q*, where *F* is the Faraday constant, assuming the four electrons are needed to produce one oxygen molecule.

### DFT calculation

Calculations were carried out with DFT implanted in the Vienna Ab initio Simulation Package (VASP)^39^,^40^,^41^,^42^ to give a better understanding for the superior activity of Ni_0.75_V_0.25_-LDH. Perdew-Burke-Ernzerhof (PBE)^43^ exchange-correlation functional and projector augmented-wave (PAW)^44^ pseudo-potential were adopted. An energy cutoff of 400 eV was applied for the plane-wave basis set. A 2 × 2 supercell was used with one of the Ni atom substituted by Fe or V. A 7 × 7 Monkhorst–Pack K-point grid was applied for the sampling of Brillouin zone. To describe the transition metal elements, DFT+U^45^ method have been used with the U values from ref. 35, U−J=3.8 eV for Ni^2+^ and U−J=4.3 eV for Fe^3+^ and U−J=3.4 eV for V^3+^ species. One proton of the material is removed to create the metal ion at 3+ oxidation state. The structures were optimized until the maxima force on the atoms was smaller than 0.02 eV/Å.

### Data availability

The data that support the findings of this study are available from the corresponding author upon request.

## Additional information

**How to cite this article**: Fan, K. *et al.* Nickel–vanadium monolayer double hydroxide for efficient electrochemical water oxidation. *Nat. Commun.* 7:11981 doi: 10.1038/ncomms11981 (2016).

## Supplementary Material

Supplementary Information Supplementary Figures 1-9, Supplementary Table 1 and Supplementary References.
