# Supplementary material for: Nickel–vanadium monolayer double hydroxide for efficient electrochemical water oxidation
Source: Nat Commun. 2016 Jun 16;7:11981. doi: 10.1038/ncomms11981 (PMC4912661; doi:10.1038/ncomms11981)
Supplement: Supplementary Information — Supplementary Figures 1-9, Supplementary Table 1 and Supplementary References. [file ncomms11981-s1.pdf]

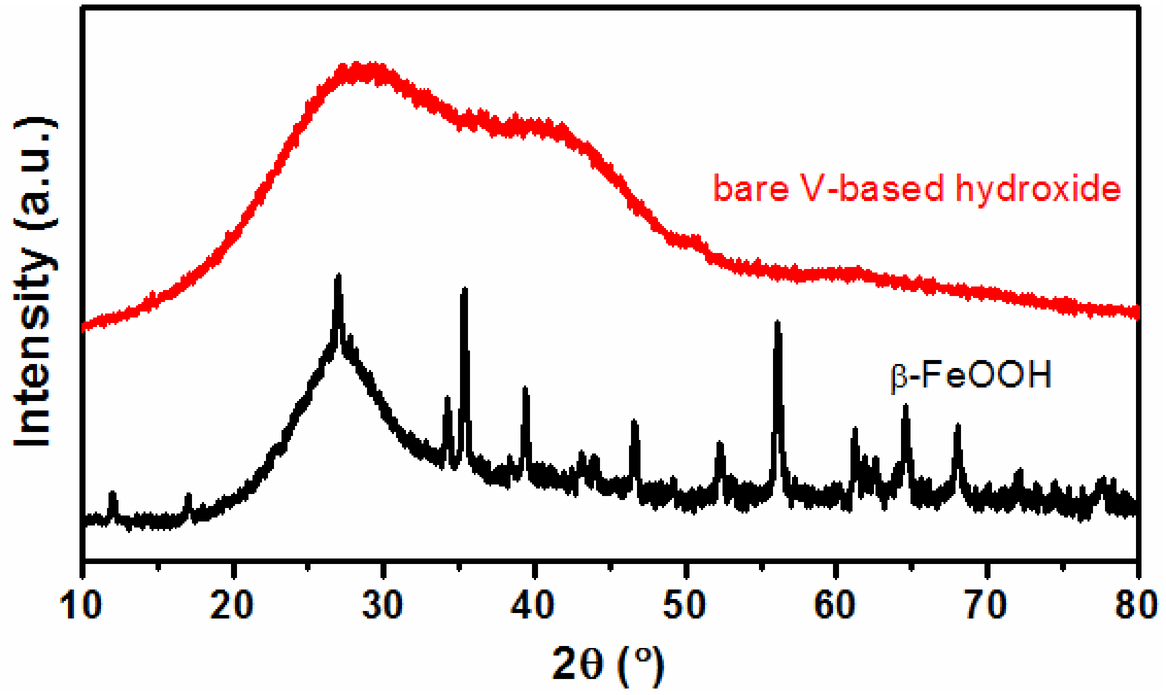

Supplementary Figure 1 | XRD pattern of as-synthesized bare V-based hydroxide and bare Fe-based hydroxide. The bare V-based hydroxide shows very low crystallinity and the XRD of bare Fe-based hydroxide indicates it is β-FeOOH.

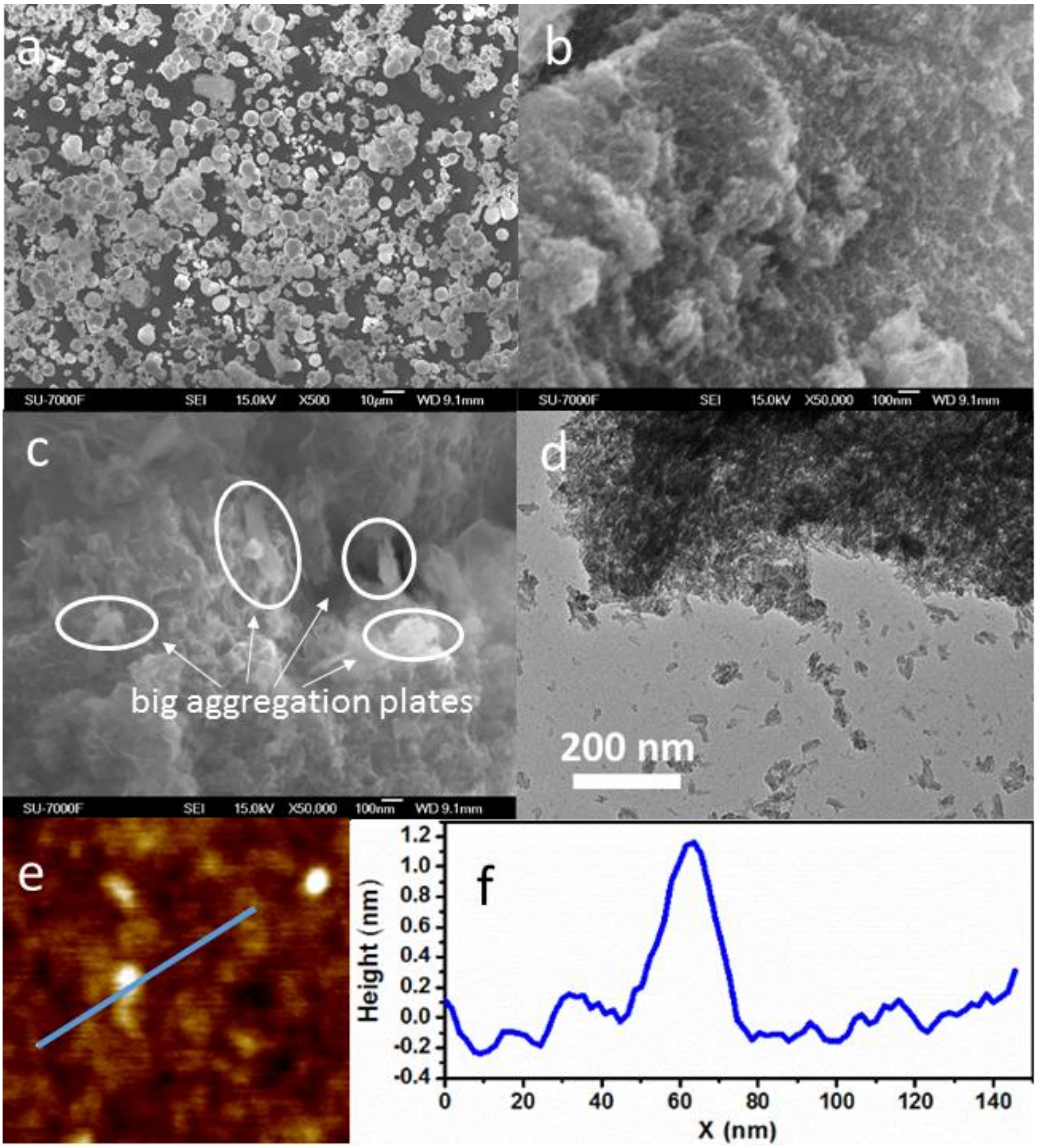

Supplementary Figure 2 | SEM, TEM and AFM of LDHs. (**a**) SEM images of as-synthesized pure α-$Ni(OH)_2$ spheres, (**b**) $Ni_{0.75}V_{0.25}$-LDH and (**c**) $Ni_{0.75}Fe_{0.25}$-LDH; (**d**) TEM (**e**) AFM and (**f**) height profile of $Ni_{0.75}Fe_{0.25}$-LDH.

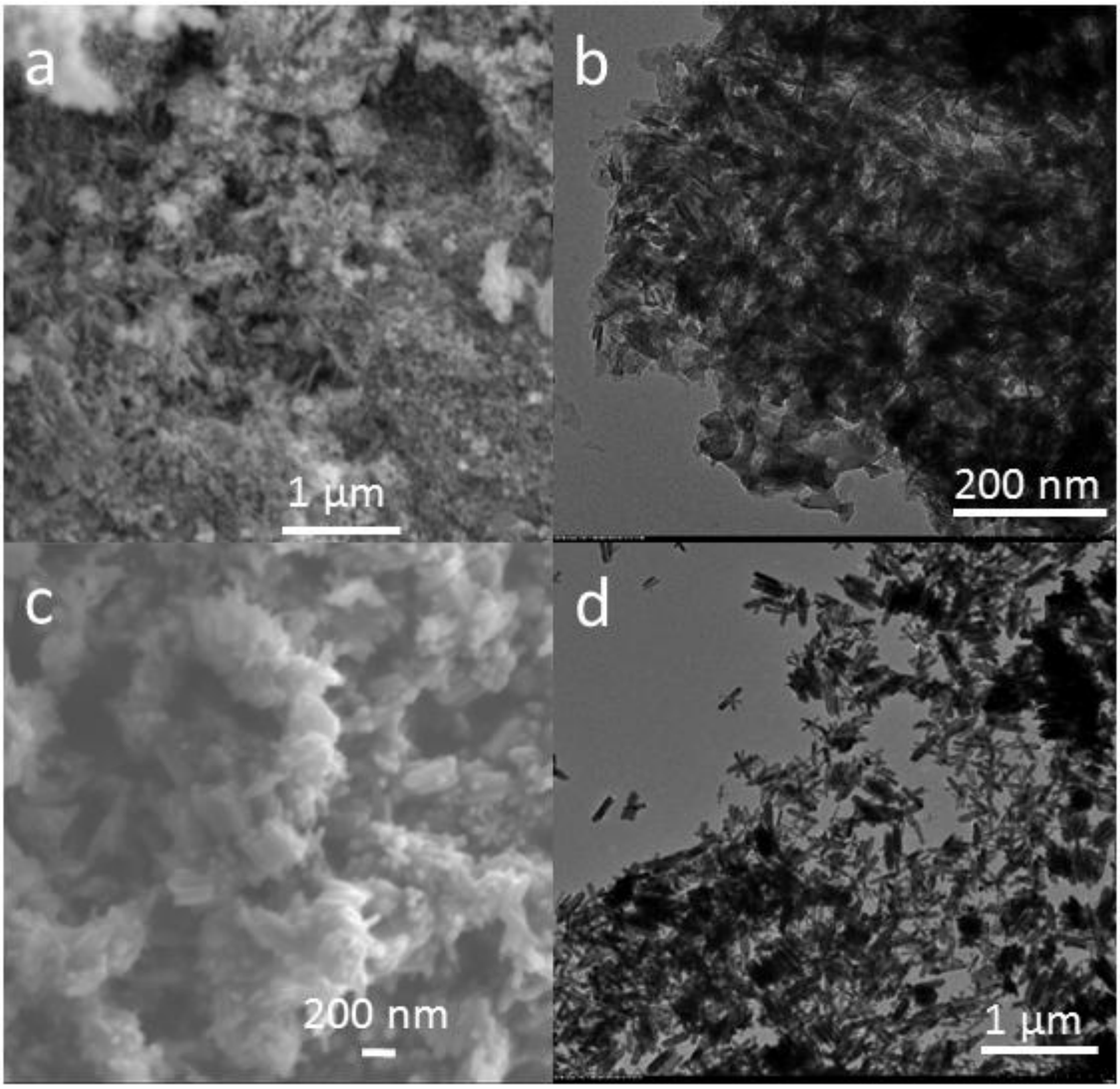

Supplementary Figure 3 | SEM and TEM of V- and Fe-based hydroxides. (**a**) SEM and (**b**)TEM images of as-synthesized bare V-based hydroxide; (**c**) SEM and (**d**)TEM images of bare Fe-based hydroxide (*β*-FeOOH).

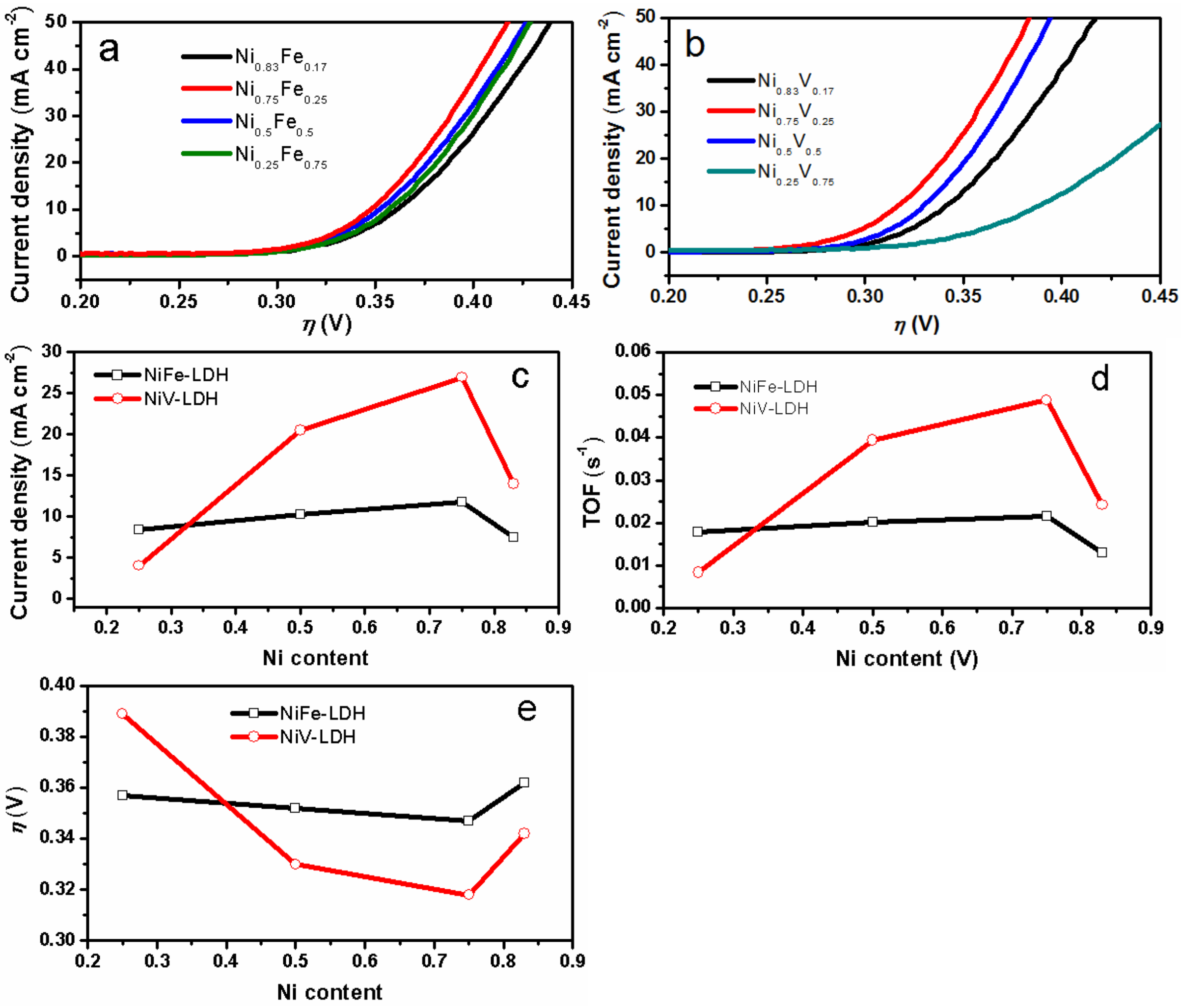

Supplementary Figure 4 | Catalytic properties of LDHs. (**a**) LSV curves of NiFe-LDHs and (**b**) NiV-LDHs with different Ni contents; (**c**) Current density and (**d**) TOF at 350 mV overpotential of NiFe-LDHs and NiV-LDHs with different Ni contents; (**e**) Overpotential required for 10 mA cm^{-2} current density of NiFe-LDHs and NiV-LDHs with different Ni contents. All the data were collected without ohmic-drop correction.

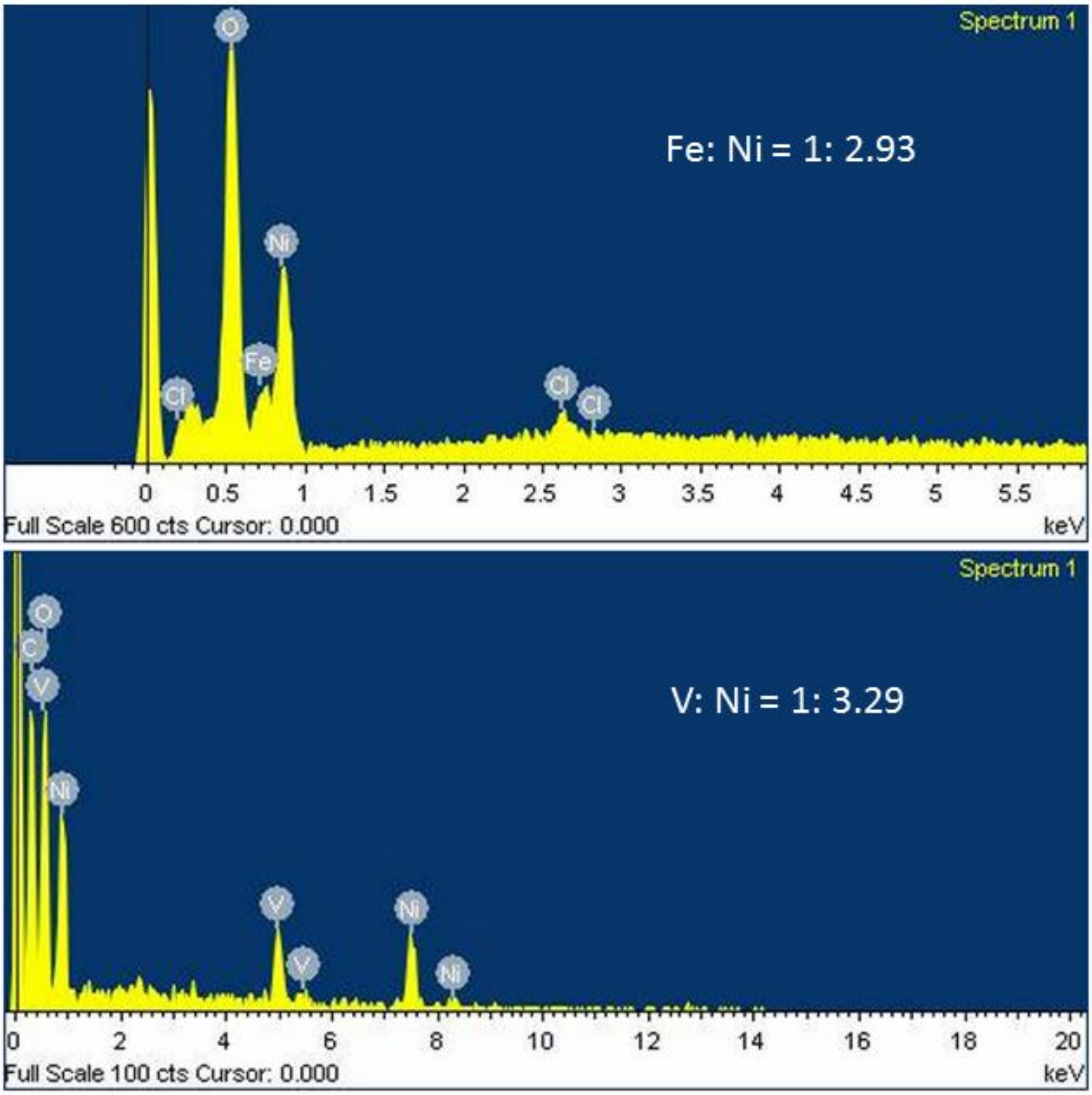

Supplementary Figure 5 | EDS. (**upper**) EDS of $Ni_{0.75}Fe_{0.25}$-LDH; (**bottom**) EDS of $Ni_{0.75}V_{0.25}$-LDH.

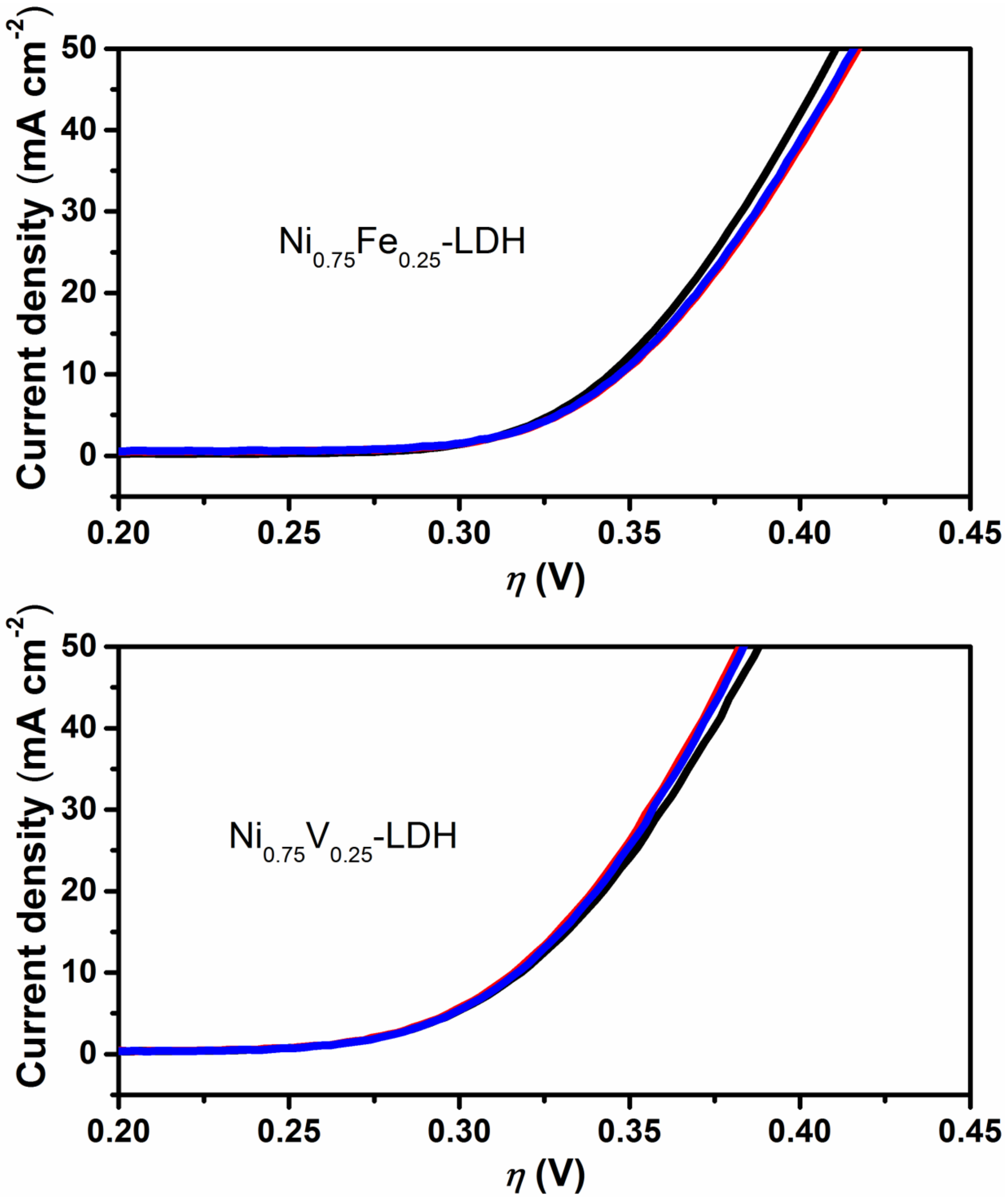

Supplementary Figure 6 | Reproducibility of water oxidation of LDH catalysts. (**upper**) Reproducibility of $Ni_{0.75}Fe_{0.25}$-LDH; (**bottom**) Reproducibility of $Ni_{0.75}V_{0.25}$-LDH. Every experiment was repeated 3 times.

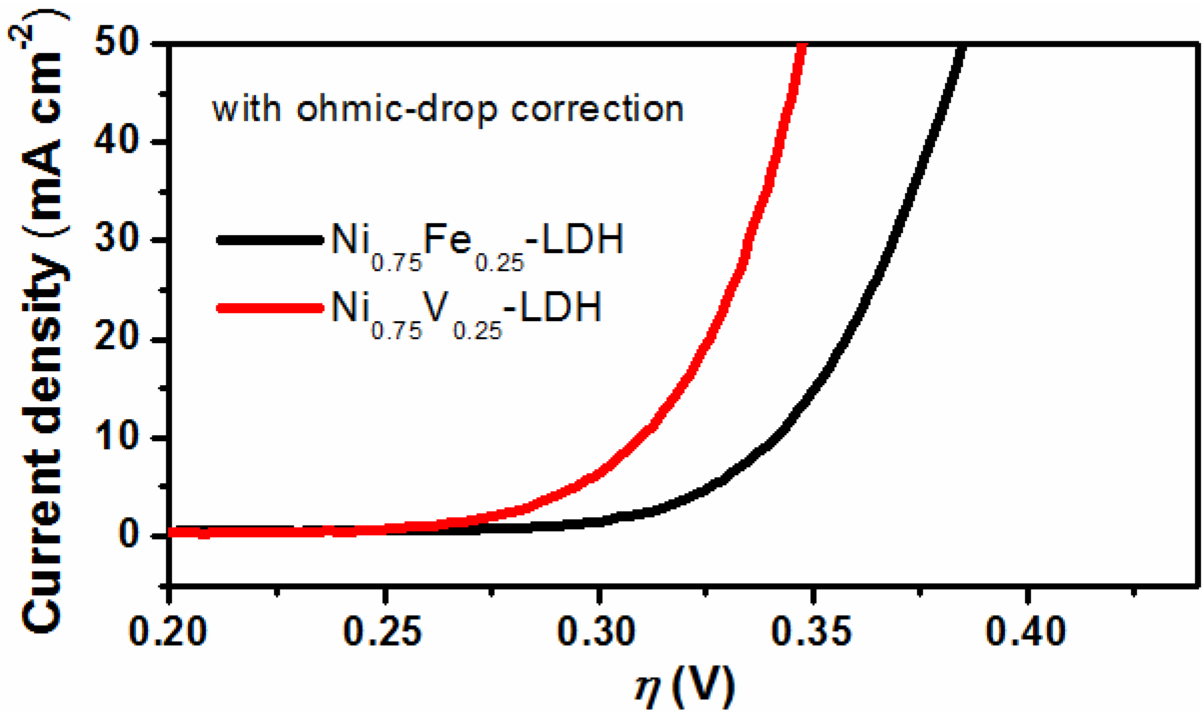

Supplementary Figure 7 | LSV curves with ohmic-drop correction of $Ni_{0.75}Fe_{0.25}$-LDH and $Ni_{0.75}V_{0.25}$-LDH. The resistance used here is around 10 Ω.

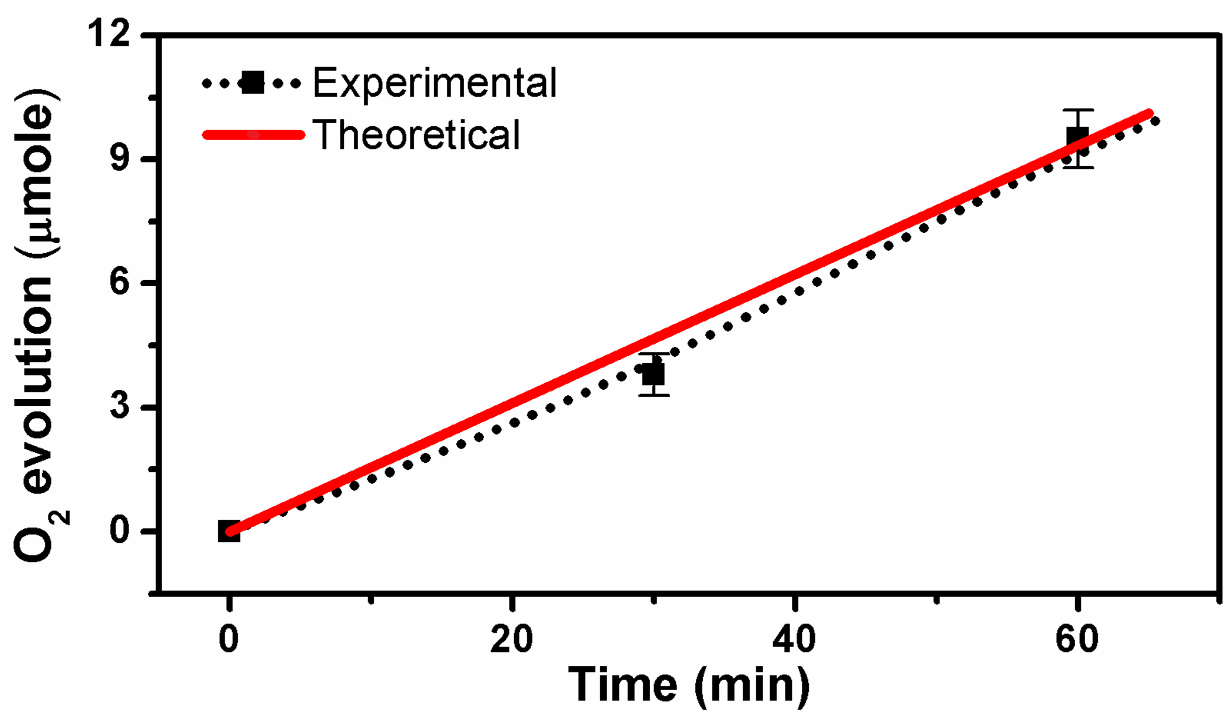

Supplementary Figure 8 | O_2 evolution. The experimental and theoretical O_2 evolution amount by $Ni_{0.75}V_{0.25}$-LDH at a constant oxidative current of 1 mA. All the error bars represent the standard deviations of three replicate measurements.

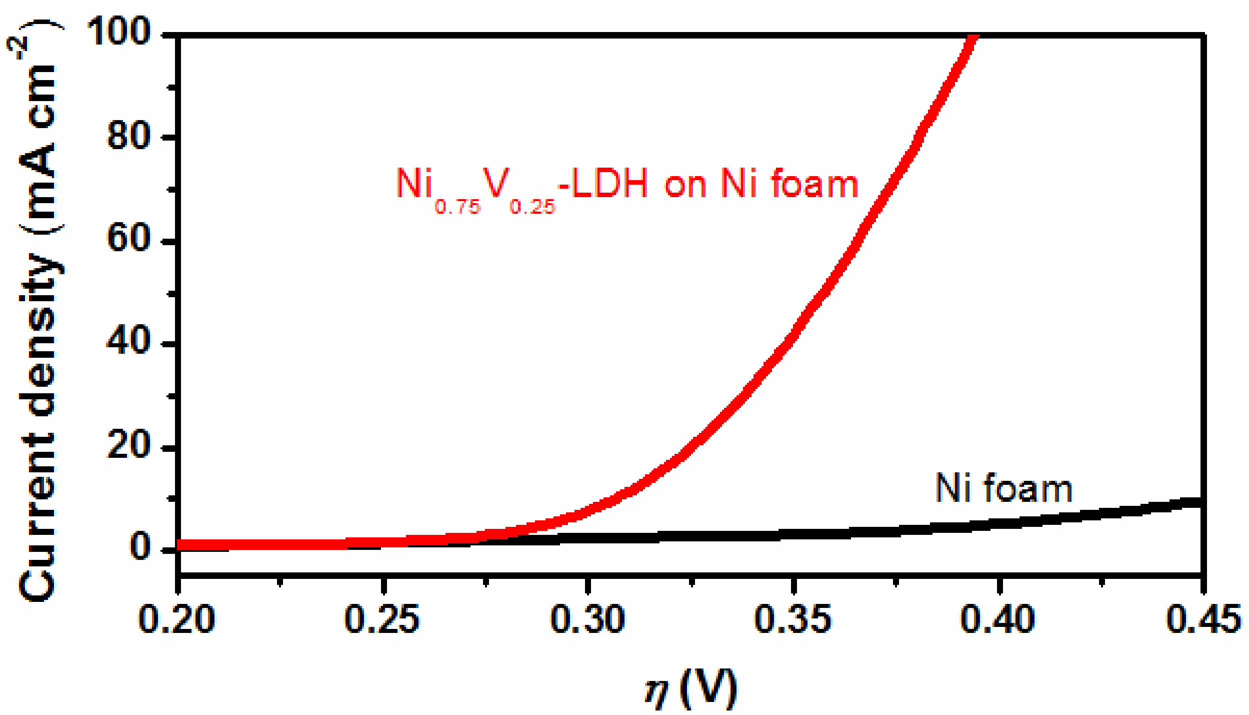

Supplementary Figure 9 | LSV curves of bare Ni foam and $Ni_{0.75}V_{0.25}$-LDH on Ni foam.

The catalyst loading amount is 0.25 mg cm^{-2}.

Supplementary Table 1 | Comparison of LDH catalysts for water oxidation. All the LDHs were loaded on GC electrode unless noted otherwise.

**LDH catalysts loaded on glassy carbon**	**Electrolyte**	**Current density (mA cm^{-2}) at η=350 mV**	**Mass activity (A g^{-1}) at η=350 mV**	**References**
NiV	1 M KOH	~ 27	~ 190	This work
NiV*	1 M KOH	~ 57	~ 400	This work
NiFe*	1 M KOH	~ 11	~ 154	1
NiFe* (exfoliated)	1 M KOH	~ 17	~ 68	2
NiFe*	1 M KOH	~ 17	~ 85	3
NiFe*	0.1 M KOH	~ 10 (η=300 mV)	-	4
NiCo*	1 M KOH	~ 3.2	~ 44.8	1
NiCo double hydroxide nanocage*	1 M KOH	~ 10	~ 49	5
NiCo (exfoliated) [a]	1 M KOH	~ 5.8	-	6
ZnCo nanosheet	0.1 M KOH	~ 1.3	-	7
ZnCo nanoparticle	0.1 M KOH	~ 0	~ 0	7
ZnCo	0.1 M KOH	<1	<3.5	8
ZnCo [b]	0.1 M KOH	~ 0	~ 0	9
CoCo*	1 M KOH	~ 2.2	~ 30.8	1
CoCo * (exfoliated)	1 M KOH	~ 11	~ 154	1
CoMn*	1 M KOH	~ 43	~ 301	10
IrO_2*	1 M KOH	~ 16	~ 112	10

* Ohmic-drop correction, [a] loaded on carbon paper, [b] loaded on Ni foil

Supplementary references

1. Song, F. & Hu, X. Exfoliation of layered double hydroxides for enhanced oxygen evolution catalysis. *Nat. Commun.* **5**, 4477 (2014).
2. Ma, W. et al. A Superlattice of Alternately Stacked Ni–Fe Hydroxide Nanosheets and Graphene for Efficient Splitting of Water. *ACS Nano* **9**, 1977-1984 (2015).
3. Gong, M. et al. An Advanced Ni–Fe Layered Double Hydroxide Electrocatalyst for Water Oxidation. *J. Am. Chem. Soc.* **135**, 8452-8455 (2013).
4. Louie, M.W. & Bell, A.T. An Investigation of Thin-Film Ni–Fe Oxide Catalysts for the Electrochemical Evolution of Oxygen. *J. Am. Chem. Soc.* **135**, 12329-12337 (2013).
5. Nai, J. et al. Efficient Electrocatalytic Water Oxidation by Using Amorphous Ni-Co Double Hydroxides Nanocages. *Adv. Energy. Mater.* **5**, doi: 0.1002/aenm.201401880 (2015).
6. Liang, H. et al. Hydrothermal Continuous Flow Synthesis and Exfoliation of NiCo Layered Double Hydroxide Nanosheets for Enhanced Oxygen Evolution Catalysis. *Nano Lett.* **15**, 1421-1427 (2015).
7. Qiao, C. et al. One-step synthesis of zinc–cobalt layered double hydroxide (Zn–Co-LDH) nanosheets for high-efficiency oxygen evolution reaction. *J. Mater. Chem. A* **3**, 6878-6883 (2015).
8. Zou, X., Goswami, A. & Asefa, T. Efficient Noble Metal-Free (Electro)Catalysis of Water and Alcohol Oxidations by Zinc–Cobalt Layered Double Hydroxide. *J. Am. Chem. Soc.* **135**, 17242-17245 (2013).
9. Li, Y., Zhang, L., Xiang, X., Yan, D. & Li, F. Engineering of ZnCo-layered double hydroxide nanowalls toward high-efficiency electrochemical water oxidation. *J. Mater. Chem. A* **2**, 13250-13258 (2014).

10. Song, F. & Hu, X. Ultrathin Cobalt–Manganese Layered Double Hydroxide Is an Efficient Oxygen Evolution Catalyst. *J. Am. Chem. Soc.* **136**, 16481-16484 (2014).
